# Using transcranial magnetic stimulation of the undamaged brain to identify lesion sites that predict language outcome after stroke

**DOI:** 10.1093/brain/awx087

**Published:** 2017-04-18

**Authors:** Diego L. Lorca-Puls, Andrea Gajardo-Vidal, Mohamed L. Seghier, Alexander P. Leff, Varun Sethi, Susan Prejawa, Thomas M. H. Hope, Joseph T. Devlin, Cathy J. Price

**Affiliations:** 1 Wellcome Trust Centre for Neuroimaging, Institute of Neurology, University College London, London WC1N 3BG, UK; 2 Department of Speech, Language and Hearing Sciences, Faculty of Health Sciences, Universidad del Desarrollo, 4070001 Concepcion, Chile; 3 Cognitive Neuroimaging Unit, Emirates College for Advanced Education, PO Box 126662 Abu Dhabi, United Arab Emirates; 4 Institute of Cognitive Neuroscience, Division of Psychology and Language Sciences, University College London, London WC1N 3AR, UK; 5 Department of Brain Repair and Rehabilitation, Institute of Neurology, University College London, London WC1N 3BG, UK; 6 Department of Neuroinflammation, Institute of Neurology, University College London, London WC1N 1PJ, UK; 7 Department of Neurology, Max Planck Institute for Human Cognitive and Brain Sciences, 04103 Leipzig, Germany; 8 Collaborative Research Centre 1052 ‘Obesity Mechanisms’, Faculty of Medicine, University of Leipzig, 04103 Leipzig, Germany; 9 Department of Experimental Psychology, Division of Psychology and Language Sciences, University College London, London WC1H 0AP, UK

**Keywords:** stroke, lesion-deficit mapping, TMS, language processing, prognosis

## Abstract

Transcranial magnetic stimulation focused on either the left anterior supramarginal gyrus or opercular part of the left inferior frontal gyrus has been reported to transiently impair the ability to perform phonological more than semantic tasks. Here we tested whether phonological processing abilities were also impaired following lesions to these regions in right-handed, English speaking adults, who were investigated at least 1 year after a left-hemisphere stroke. When our regions of interest were limited to 0.5 cm^3^ of grey matter centred around sites that had been identified with transcranial magnetic stimulation-based functional localization, phonological impairments were observed in 74% (40/54) of patients with damage to the regions and 21% (21/100) of patients sparing these regions. This classification accuracy was better than that observed when using regions of interest centred on activation sites in previous functional magnetic resonance imaging studies of phonological processing, or transcranial magnetic stimulation sites that did not use functional localization. New regions of interest were generated by redefining the borders of each of the transcranial magnetic stimulation sites to include areas that were consistently damaged in the patients with phonological impairments. This increased the incidence of phonological impairments in the presence of damage to 85% (46/54) and also reduced the incidence of phonological impairments in the absence of damage to 15% (15/100). The difference in phonological processing abilities between those with and without damage to these ‘transcranial magnetic stimulation-guided’ regions remained highly significant even after controlling for the effect of lesion size. The classification accuracy of the transcranial magnetic stimulation-guided regions was validated in a second sample of 108 patients and found to be better than that for (i) functional magnetic resonance imaging-guided regions; (ii) a region identified from an unguided lesion overlap map; and (iii) a region identified from voxel-based lesion-symptom mapping. Finally, consistent with prior findings from functional imaging and transcranial magnetic stimulation in healthy participants, we show how damage to our transcranial magnetic stimulation-guided regions affected performance on phonologically more than semantically demanding tasks. The observation that phonological processing abilities were impaired years after the stroke, suggests that other brain regions were not able to fully compensate for the contribution that the transcranial magnetic stimulation-guided regions make to language tasks. More generally, our novel transcranial magnetic stimulation-guided lesion-deficit mapping approach shows how non-invasive stimulation of the healthy brain can be used to guide the identification of regions where brain damage is likely to cause persistent behavioural effects.

## Teaser

Predicting how brain damage will affect cognition is notoriously challenging. By using findings from non-invasive brain stimulation studies of healthy subjects to guide lesion-deficit mapping, Lorca-Puls *et al*. identify two lesion sites, in frontal and parietal areas, that consistently cause persistent language difficulties after stroke.

## Introduction

Previous research has shown that repetitive transcranial magnetic stimulation (TMS) over either the left supramarginal gyrus (SMG) (Hartwigsen *et al.*, 2010*a*; Sliwinska *et al.*, 2015) or pars opercularis of the left inferior frontal gyrus (pOp) (Gough *et al.*, 2005; Hartwigsen *et al.*, 2010*b*) causes significant slowing in response times when healthy volunteers perform tasks requiring phonological processing (e.g. do two words sound the same?) relative to tasks requiring semantic processing (e.g. do two words mean the same?). These TMS findings are consistent with a wealth of functional imaging studies that have shown activation in SMG and/or pOp for phonological relative to semantic tasks (Price *et al.*, 1997; Poldrack *et al.*, 1999; Booth *et al.*, 2002; Devlin *et al.*, 2003; McDermott *et al.*, 2003; Seghier *et al.*, 2004; Gitelman *et al.*, 2005; Simard *et al.*, 2013).

In the current study, we hypothesized that, if SMG and pOp are necessary for phonological processing, then stroke damage to these regions should impair the ability to perform phonological tasks, unless other brain regions (e.g. in the right hemisphere) can compensate. More generally, if this proof-of-principle study finds that stroke damage to regions associated with phonological processing in TMS or functional MRI studies also impairs phonological processing in stroke patients, then TMS and/or functional MRI may provide a useful tool for identifying and understanding lesion sites that predict outcome after stroke.

We also make a distinction between regions of interest that were previously identified using TMS-based functional localization (Gough *et al.*, 2005; Sliwinska *et al.*, 2015) and TMS sites that were centred on areas of peak activation in previous functional MRI studies (Hartwigsen *et al.*, 2010*a*, *b*). This is because TMS-based functional localization has shown that the site that is most sensitive to TMS (averaged over subjects) does not necessarily correspond to the site of peak activation in functional MRI studies (averaged over subjects). For example, the average TMS pOp site (−52, 16, 8) identified after functional localization in Gough *et al.* (2005) is at least 1 cm anterior and inferior to the functional MRI coordinates for pOp activation (−50, 6, 24) reported in Devlin *et al.* (2003), with an Euclidean distance of 19 mm between the two points. Likewise, the average TMS SMG site (−52, −34, 30) identified after functional localization by Sliwinska *et al.* (2015) is at least 1 cm inferior and lateral to the functional MRI coordinates for SMG activation (−42, −40, 46) reported in the functional MRI study by Devlin *et al.* (2003), with an Euclidean distance of 20 mm between the two points. In contrast, the TMS sites reported by Hartwigsen *et al.* (2010*a*, *b*) without functional localization (−47, 6, 21 for pOp and −45, −39, 45 for SMG) are almost identical to those reported in the functional MRI study by Devlin *et al.* (2003).

Previous lesion studies have shown that stroke damage to SMG and/or pOp and/or the white matter underlying these areas can cause deficits in tasks that require phonological processing, including speech production (Mirman *et al.*, 2015), sentence production (Faroqi-Shah *et al.*, 2014), non-word repetition (Faroqi-Shah *et al.*, 2014), phonological decisions (Geva *et al.*, 2011), and increase the number of phonological errors produced during picture naming (Schwartz *et al.*, 2012). However, these studies (i) summed effects across groups of patients and therefore did not indicate how consistently damage causes phonological impairments in individual subjects; (ii) included patients who had large lesions affecting multiple brain regions, and therefore did not establish whether damage to SMG alone or pOp alone is sufficient to impair phonological processing; (iii) did not compare the location of the stroke lesions with the location of the sites where TMS impairs phonological processing abilities; and (iv) did not analyse the level of processing that affected performance on phonological tasks. Moreover, previous research that has specifically focused on identifying the brain areas where tissue loss is associated with phonological and semantic abilities (Butler *et al.*, 2014; Halai *et al.*, 2017) did not report the SMG or pOp sites from the TMS studies that we focus on in the current paper (Gough *et al.*, 2005; Sliwinska *et al.*, 2015). Nor did they reverse the question to ask: How consistently and persistently does damage to specific brain regions cause behavioural symptoms that are indicative of phonological impairments? The answers to such questions have important clinical implications for predicting language outcome and recovery after stroke.

To summarize, in contrast to previous studies, we sought to establish whether, and how consistently, stroke damage to the SMG and/or pOp sites identified in TMS studies that used functional localization (Gough *et al.*, 2005; Sliwinska *et al.*, 2015) impairs the ability to perform tasks that require phonological processing. We also investigated (i) whether phonological tasks were more severely affected than semantic tasks; and (ii) how the TMS sites of interest could be moved, tailored and expanded to account for more patients with impaired phonological processing abilities; while (iii) carefully controlling for lesion size when comparing performance between groups of patients with different lesion locations (Price *et al.*, 2017). Finally, we tested how well damage to these new ‘TMS-guided’ sites predicted phonological processing abilities in a second sample of 108 patients, many years after they had suffered a left-hemisphere stroke.

By selecting regions of interest from TMS studies of healthy participants, our lesion-deficit associations should not be spatially biased towards brain areas that are most susceptible to vascular events, unlike traditional univariate voxel-based lesion-symptom mapping (VLSM) techniques (Inoue *et al.*, 2014; Mah *et al.*, 2014). To demonstrate this, we compare how well phonological impairments are accounted for by lesions to our TMS-guided regions relative to lesions to other regions of interest from prior functional MRI studies, a functional MRI-guided lesion overlap map (LOM), an unguided LOM, and voxel-based lesion-symptom mapping.

Our hypothesis is that if damage to SMG or pOp causes persistent difficulties with tasks that rely on phonological processing, then the function of these regions is not completely compensated for by other brain regions. Alternatively, if damage to SMG or pOp does not impair performance on phonologically demanding tasks, then recovery after stroke might involve the function of SMG and pOp being successfully compensated for by other brain areas. TMS studies in healthy participants cannot answer these questions because they infer a region’s contribution to task efficiency on the basis of slowed response times during disruptive stimulation. In contrast, the use of lesions caused by stroke allows us to infer that a region was essential for task performance on the basis of a significant and persistent impairment of accuracy following damage to the area.

## Materials and methods

### Participants

Data from all participants were extracted from the PLORAS database (Predicting Language Outcome and Recovery After Stroke; Price *et al.*, 2010; Seghier *et al.*, 2016). At a minimum, the data available for each patient included: a full assessment of speech and language abilities using the Comprehensive Aphasia Test (CAT) (Swinburn *et al.*, 2004); and a 3D lesion image, in standard space, created from a T_1_-weighted high resolution (1 mm isotropic voxels) anatomical whole-brain volume, using our automated lesion identification software (Seghier *et al.*, 2008). The study was approved by the London Queen Square Research Ethics Committee. All patients gave written informed consent prior to participation and were compensated £10 per hour for their time.

We report data from a total of 288 adult stroke survivors who were all native speakers of English with normal or corrected-to-normal vision and hearing. All were right-handed (pre-morbidly) with no history of neurological or psychiatric illness that was not related to their stroke. These 288 patients were split into three main samples as follows:

Sample 1 comprised 154 patients (43 females) who were assessed between 1 to 5 years after a left-hemisphere stroke [mean = 2.7 years, standard deviation (SD) = 1.2] that was greater than 1 cm^3^ (mean = 80.1 cm^3^, SD = 79.9, range = 1.4–464.7). Their mean age was 59 years (SD = 12.7, range = 21.3–90.0). This group was used to test how consistently damage to different regions of interest was associated with phonological impairments; and how the regions of interest could be adapted to provide the best account of the data.

Within Sample 1, we selected two subsets of patients to determine the percentage of damage to different regions of interest that best accounted for the presence or absence of phonological impairments. Sample 1A included those who were categorized (using the criteria described in the next section) with phonological impairments but not semantic impairments. Sample 1B included those who were matched to Sample 1A for left-hemisphere lesion size but did not meet the criteria we used to define phonological impairments. Lesion size was matched between the two groups by finding the minimum and maximum lesion volumes that were common to both groups with no significant differences in mean lesion size across groups. The resulting group size was 23 patients (seven females) in Sample 1A and 32 patients (two females) in Sample 1B; lesion size mean and range = 82.2 cm^3^, 44.3–128.7 cm^3^ in Sample 1A and 76.0 cm^3^, 44.0–135.7 cm^3^ in Sample 1B. The groups were also matched for age (mean = 54 and 58 years; range = 21.3–78.2 and 29.4–76.1) and time post-stroke (mean = 2.4 and 3.0 years; range = 1.0–4.5 and 1.1–4.7).

Sample 2 comprised 108 patients (mean age = 61 years, SD = 11.0, range = 33.2–83.6; 42 females) who differed from those in Sample 1 because they were assessed more than 5 years after a left-hemisphere stroke (mean = 10.1 years, SD = 6.0, range = 5.1–36.0). As in Sample 1, lesion size was always >1 cm^3^ (mean = 129.3, SD = 106.5, range = 1.2–405.0). This sample was used to validate the lesion-deficit associations identified with Sample 1, while also considering the effect of time post-stroke (>5 years versus 1–5 years).

Sample 3 comprised 42 patients (15 females) who, in addition to having the standard PLORAS assessments were also tested on the phonological and semantic decision tasks used in the TMS studies from which our regions of interest were derived. Comparison of their performance on the TMS and CAT tasks allowed us to select the CAT tasks that best captured the variance in the TMS phonological task. Their mean age was 63 years (SD = 12.3, range = 28.0–84.0) and the mean time they were tested after a stroke to the left hemisphere (18 patients), right hemisphere (22 patients) or both (two patients) was 9.7 years (SD = 6.9, range = 1.1–35.1). Their mean lesion size was 76.4 cm^3^ (SD = 79.1, range = 3.7–302.8). There was no overlap between Samples 1 and 3 (because none of the patients in Sample 3 met the criteria for Sample 1). The overlap between Samples 2 and 3 was 16 patients.

### Defining phonological and semantic impairments

**Figure 1:**
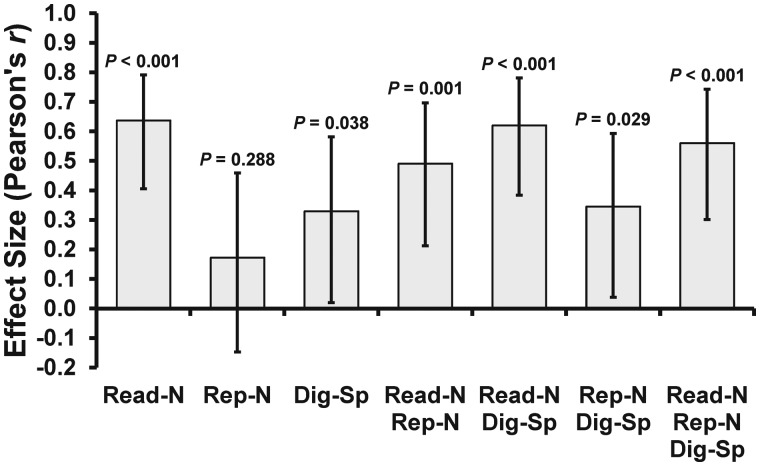
**Correlation coefficients for the CAT and TMS phonological measures.** The TMS phonological measure was the homophone judgement task used by Gough *et al.* (2005) and Sliwinska *et al.* (2015). Scores on this task (i.e. per cent accuracy ÷ median correct reaction time, × 1000) were correlated with seven different phonological measures from the CAT: non-word reading (Read-N), non-word repetition (Rep-N), digit span (Dig-Sp), and every combination of them (T-scores were averaged for each task pair/triplet). Two patients were classified as outliers because they had scores >3 SD below the group mean on the non-word reading task and were therefore removed from all correlation analyses. Error bars represent 95% CI.

To assess phonological processing independently of semantics, we selected tasks from the CAT (Swinburn *et al.*, 2004) that required phonological processing with minimum demands on semantics. These are ‘non-word reading’, ‘non-word repetition’ and ‘digit span’. Figure 1 shows how scores on these three tasks, and every possible combination of them, correlated with scores on the TMS phonological task in the 42 patients in Sample 3 who were tested on both the CAT and TMS tasks (Gough *et al.*, 2005; Sliwinska *et al.*, 2015). The CAT measures that were most strongly correlated with the TMS phonological task were non-word reading [*r*(40) = 0.64] and the combination of non-word reading and digit span [*r*(40) = 0.62]. Both these phonological measures were also significantly more correlated with the TMS phonological task than the TMS semantic task (*z* = 2.89, *P* = 0.004 and *z* = 3.00, *P* = 0.003, respectively).

We chose to use the combined non-word reading and digit span measure because (i) impaired phonological processing should affect both tasks; and (ii) an impairment on non-word reading but not digit span could arise from a non-phonological level (e.g. visual perceptual/orthographic processing level) (see Supplementary Fig. 1 for our task analysis). The contribution of deficits in perceptual processing (either visual or auditory) was also controlled by showing that performance was significantly worse on our combined phonological measure than three semantic picture matching tasks (see below for task details). Finally, to assess whether phonological processing impairments were at the level of overt or covert speech production, we also report the results of a writing task that required patients to silently write down the words they have heard. This involves phonological processing without overt speech production.

Although the phonological and semantic tasks were different in this lesion study to the tasks used to define the regions in the TMS studies, a cross task validation ensures that the function of the regions being investigated is involved in the computation of phonological processes that are shared by a range of different language tasks. It is also more feasible to generalize over tasks because of the challenges of using the CAT speech production tasks in a TMS study and, conversely, the TMS phonological tasks in the stroke patient study. Specifically, speech response times for the CAT tasks are difficult to measure in TMS studies because voice onset times are hidden by noise and jaw movements. Conversely, the problem administering the TMS phonological tasks to patients is that most patients with post-stroke aphasia do not generate enough correct responses that can be used to measure response times.

### Task details

The CAT non-word reading task visually presents five nonsense words (e.g. fask), one at a time, with instructions to read them aloud. Immediate correct responses were given a score of 2; incorrect responses were given a score of 0; correct responses after a self-correction or a delay (>5 s) were given a score of 1. Articulatory errors (e.g. dysarthric distortions) not affecting the perceptual identity of the target were scored as correct. Verbal, phonemic, neologistic and apraxic errors were scored as incorrect. T-scores ≤56 constitute the impaired range.

The CAT digit span task involves hearing digit strings and repeating what has been heard. There are six progressive levels of difficulty that start with two digits and build up to seven digits. The total score is obtained by multiplying the number of digits in the digit string of maximum length successfully repeated by two. Phonemic, apraxic and dysarthric errors were not penalized. T-scores ≤50 constitute the impaired range.

The CAT writing heard words task involved hearing five words (one at a time) and writing them down as accurately as possible. The test items comprise a concrete word ‘man’, an irregular concrete word ‘yacht’, an abstract word ‘idea’, a morphologically complex word ‘undrinkable’, and a non-word ‘blosh’. Letters in the correct position were given a score of 1 each. Substitutions, omissions and transpositions were given a score of 0. One point was deducted from the total score if one or more letters were added to the target word. T-scores ≤57 constitute the impaired range.

The CAT visual word-to-picture matching task involves a written word at the centre of the page surrounded by four possible pictures. The subject has to pick the picture that matches the meaning of the written word. There are a total of 15 test trials plus a practice one at the beginning. The scoring system for this task was identical to that used in the non-word reading task. T-scores ≤53 constitute the impaired range.

The CAT auditory word-to-picture matching task involves hearing a word produced by the examiner and selecting the picture among four possible alternatives that best matches the meaning of the heard word. There are a total of 15 test trials plus a practice one at the beginning. The scoring system for this task was identical to that used in the non-word reading task. T-scores ≤51 constitute the impaired range.

The CAT semantic associations task presented five pictures of objects. The instructions were to match the picture at the centre (e.g. mitten) with one of four possible alternatives according to the strongest semantic association (e.g. hand, sock, jersey, and lighthouse). The inclusion of a semantically related distractor (e.g. sock) encouraged deeper levels of semantic processing. There are a total of 10 test trials plus a practice one at the beginning. Correct responses were given a score of 1; incorrect responses were given a score of 0. T-scores ≤47 constitute the impaired range.

Each patient was assigned a total score for every task they completed as part of the CAT assessment. For ease of comparison across tasks, standardized procedures from the CAT convert these raw scores into T-scores, which represent each patient’s assessed skill on each task relative to a reference population of 113 aphasic patients, 56 of whom were tested more than once on the CAT. The threshold for impairment is defined relative to a second population of 27 neurologically normal controls, as the point below which the score would place the patient in the bottom 5% of the control population (Swinburn *et al.*, 2004). Lower scores indicate poorer performance.

### MRI data acquisition, preprocessing and lesion identification

T_1_-weighted high resolution anatomical whole-brain volumes were obtained for all patients (*n* = 288). Four different MRI scanners (Siemens Healthcare) were used to acquire the structural images: 150 patients were imaged on a 1.5 T Sonata scanner, 115 on a 3 T Trio scanner, 18 on a 1.5 T Avanto scanner, and five on a 3 T Allegra scanner. As for the 1.5 T Avanto scanner, a 3D magnetization-prepared rapid acquisition gradient-echo (MPRAGE) sequence was used to acquire 176 sagittal slices with a matrix size of 256 × 224, yielding a final spatial resolution of 1 mm isotropic voxels (repetition time/echo time/inversion time = 2730/3.57/1000 ms). In all other cases, an optimized 3D modified driven equilibrium Fourier transform (MDEFT) sequence was used to acquire 176 sagittal slices with a matrix size of 256 × 224, yielding a final spatial resolution of 1 mm isotropic voxels: repetition time/echo time/inversion time = 12.24/3.56/530 ms and 7.92/2.48/910 ms at 1.5 T and 3 T, respectively (Deichmann *et al.*, 2004). The structural image for each patient was then converted to a 3D binary lesion image (specifying where damage was likely in the brain, in comparison to healthy controls), in standard MNI space, using our automated lesion identification procedure that has been described in full elsewhere (Seghier *et al.*, 2008).

### Regions of interest from prior TMS and functional MRI studies

**Figure 2:**
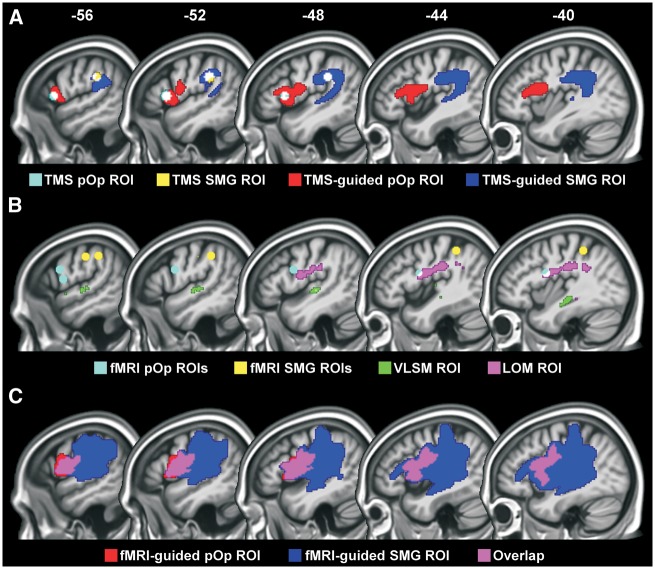
**Regions of interest.** (**A**) The TMS and TMS-guided regions are shown in cyan and red for pOp, with the 80% overlap of these regions in white; and yellow and blue for SMG, with the 82% overlap of these regions in white. (**B**) Other regions of interest are shown in cyan (functional MRI pOp), yellow (functional MRI SMG), green (VLSM) and violet (unguided LOM), with areas of overlap in white. (**C**) The functional MRI-guided regions are shown in red for pOp and blue for SMG, with areas of overlap in violet. fMRI = functional MRI; ROI = region of interest.

Our TMS regions of interest were spheres (radius of 5 mm, 0.5 cm^3^ in volume), centred on the mean MNI coordinates for SMG [−52, −34, 30] and pOp [−52, 16, 8] that were reported in studies that used TMS-based functional localization (Gough *et al.*, 2005; Sliwinska *et al.*, 2015) (Fig. 2A). The size of the regions (5 mm radius) was chosen based on the expected spatial resolution of TMS, which has been argued to be in the order of 5–10 mm (Brasil-Neto *et al.*, 1992*a*,*b*; Wilson *et al.*, 1993; Ravazzani *et al.*, 1996; Thielscher and Kammer, 2002). We also investigated how our results would change if our regions of interest had been based on previous functional MRI studies rather than previous TMS studies. The functional MRI *x*, *y*, *z* MNI coordinates for SMG were [−57, −21, 39; −42, −40, 46; −54, −36, 40; mean = −51, −32, 42] from Booth *et al.* (2002), Devlin *et al.* (2003), and Seghier *et al.* (2004), respectively (Fig. 2B). Those for pOp were [−58, 5, 13; −50, 6, 24; −57, 9, 24; −41, 3, 20; mean = −52, 6, 20] from McDermott *et al.* (2003), Devlin *et al.* (2003), Gitelman *et al.* (2005), and Simard *et al.* (2013), respectively (Fig. 2B). The coordinates reported in McDermott *et al.* (2003) and Seghier *et al.* (2004) were converted from Talairach space to MNI space using the tal2icbm transformation (Lancaster *et al.*, 2007).

### Regions of interest from data-driven analyses

Four types of regions were generated to best account for the presence or absence of phonological impairments according to the presence or absence of damage: (i) TMS-guided regions; (ii) functional MRI-guided regions; (iii) an unguided-LOM region from a LOM that was independent of the TMS regions; and (iv) a VLSM region from a VLSM analysis.

The TMS-guided regions were based on two different LOMs (from patients in Sample 1). One for those who had phonological impairments following above-threshold damage (see below for critical damage thresholds) to the 0.5 cm^3^ TMS SMG region and one for those who had phonological impairments following above-threshold damage (see below for critical damage thresholds) to the 0.5 cm^3^ TMS pOp region. The new TMS-guided regions included the grey and white matter, around the original TMS sites, that was most consistently damaged in the LOMs. For pOp, the new region extended to 20.6 cm^3^ and was 100% damaged in 12/13 of the patients with lesions to the original TMS pOp region and phonological impairments. For SMG, the new region extended to 24.9 cm^3^ and was at least 85% damaged in 11/12 of the patients with lesions to the original TMS SMG region and phonological impairments (Fig. 2A and Supplementary Fig. 2).

The functional MRI-guided regions were identified in the same way as the TMS-guided regions, with the only exception that patients (from Sample 1) with damage to spherical regions of interest centred on the mean functional MRI coordinates (−52, 6, 20 for pOp and −51, −32, 42 for SMG) were used to create the LOMs. For pOp, the new region extended to 18.1 cm^3^ and was 100% damaged in 17/17 of the patients with lesions to the original functional MRI pOp region and phonological impairments (Fig. 2C). For SMG, the new region was based on one patient only, because no other patients with phonological impairments had selective damage to the original functional MRI SMG region. Consequently, the new SMG region was very large (161.8 cm^3^) and encompassed most of the left perisylvian cortex, including 94% of the functional MRI-guided pOp region (Fig. 2C). Therefore, this additional region added no explanatory power.

The unguided-LOM region was based on a single (unguided) overlap map of the lesion images from all 23 patients in Sample 1A (with phonological but not semantic impairments). The purpose of this analysis was to identify brain regions commonly damaged in patients with selective phonological impairments. We found that the maximum number of patients who had damage at any given voxel was 19 (out of 23). This degree of overlap, however, was only observed in a very small region (i.e. 0.1 cm^3^). Comparison of the classification accuracy for different degrees of overlap identified that an overlap of 16 patients (out of 23), observed over a 7.3 cm^3^ region (Fig. 2B), was the best predictor of performance.

Finally, the VLSM region was based on a univariate VLSM analysis (Bates *et al.*, 2003; Rorden *et al.*, 2007) that identified voxels where the frequency of lesions was significantly greater in the 23 patients with phonological but not semantic impairments (Sample 1A) than the 32 patients who did not meet the criteria for phonological impairments (Sample 1B). This analysis was conducted using procedures described in Rorden *et al.* (2007) including limiting the analysis to voxels that were damaged in at least 20% of the 55 patients (as in Geva *et al.*, 2011). The resulting VLSM region of interest (1.0 cm^3^) comprised all the voxels that surpassed a statistical threshold of *P* < 0.001 uncorrected, one-tailed (Fig. 2B). We obtained virtually the same result when we searched for regions where tissue loss correlated with our phonological scores in a single group analysis that combined Samples 1A and 1B. Neither the two-group nor one-group analyses identified effects that were significant after correction for multiple comparisons (i.e. *P* < 0.05, FWE-corrected).

### Determining the threshold for critical damage

The degree to which any region was damaged varied across patients from 100% to 0%. For each region, we searched for the percentage of damage that best accounted for the presence or absence of phonological impairments in Samples 1A and 1B. Classification accuracy (for each threshold) was expressed in terms of positive and negative predictive values, sensitivity, specificity and the odds ratio (Altman and Bland, 1994*a*, *b*; Bland and Altman, 2000; Glas *et al.*, 2003). The threshold was set at the degree of damage that had the highest odds ratio.

### Other analyses

Having identified different regions and their critical damage thresholds, we compared the classification accuracy (using the odds ratio) for each region. The incidence and severity of impairments on phonologically and semantically demanding tasks was also compared. All statistical analyses of behaviour were conducted in IBM SPSS Statistics for Windows, Version 22.0 (Armonk, NY: IBM Corp). The comparison of predictive values between different regions was performed using the method proposed by Leisenring *et al.* (2000) as implemented in the R package ‘DTComPair’ (Stock and Hielscher, 2014).

## Results

The thresholds for the degree of damage to each of our six sets of regions (TMS, functional MRI, TMS-guided, functional MRI-guided, unguided-LOM, and VLSM) that best explained the presence or absence of phonological impairments was: 80%, 80%, 80%, 90%, 90% and 70%, respectively (Table 1). Henceforth, we refer to ‘damage’ when the degree of damage to the region of interest was equal to or greater than the threshold selected for that region.

**Table 1:** Classification accuracy for each region of interest

Region of interest	Threshold, %	*n*	PPV, %	NPV, %	Sensitivity, %	Specificity, %	Odds ratio
**Sample 1 (1–5 years post-stroke), regions of interest from previous studies (independent of data)**							
(1) TMS	100	55	25	57	4	91	0.4
	90	55	47	60	30	75	1.3
	**80**	55	57	68	52	72	**2.8**
	70	55	48	63	52	59	1.6
	All at 80	154	74	79	66	85	**10.7**
(2) Functional MRI	100	55	29	54	17	69	0.5
	90	55	41	57	48	50	0.9
	**80**	55	45	64	65	44	**1.5**
	70	55	42	58	65	34	1.0
	All at 80	154	64	80	72	73	**7.0**
(1) and (2)	80	154	64	84	80	70	9.5
**Sample 1 (1–5 years post-stroke), data-driven regions of interest**							
(3) TMS-guided	100	55	70	64	30	91	4.2
	90	55	69	67	39	88	4.5
	**80**	55	70	74	61	81	**6.7**
	70	55	54	70	65	59	2.7
	All at 80	154	85	85	75	91	**32.6**
(4) Functional MRI-guided	100	55	100	60	9	100	–
	**90**	55	67	61	17	94	**3.2**
	80	55	57	60	17	91	2.0
	70	55	55	61	26	84	1.9
	All at 90	154	88	73	46	96	**18.9**
(5) Unguided-LOM	100	55	100	63	17	100	–
	**90**	55	70	64	30	91	**4.2**
	80	55	56	65	43	75	2.3
	70	55	52	67	57	63	2.2
	All at 90	154	89	76	54	96	**26.2**
(6) VLSM	100	55	75	61	13	97	4.7
	90	55	67	63	26	91	3.4
	80	55	69	67	39	88	4.5
	**70**	55	71	68	43	88	**5.4**
	All at 70	154	85	76	54	94	**17.1**
(3) and (4)	80/90	154	85	86	77	91	35.7
(3) and (5)	80/90	154	83	87	80	89	33.9
(3) and (6)	80/70	154	83	91	87	88	49.4
**Sample 2 (>5 years post-stroke), regions of interest from Sample 1 (independent of data)**							
(3)	80	108	64	94	93	69	**27.6**
(4)	90	108	65	76	55	82	5.7
(5)	90	108	72	81	65	85	10.8
(6)	70	108	66	75	53	84	5.7
(3) and (4)	80/90	108	64	94	93	69	27.6
(3) and (5)	80/90	108	63	96	95	68	39.7
(3) and (6)	80/70	108	59	95	95	62	30.7

*n* = number of patients included in each analysis (55 = 23 + 32 from Samples 1A and 1B; 154 = all those 1–5 years post-stroke; 108 = all those >5 years post-stroke). PPV/NPV = positive and negative predictive values. Threshold = cut-off (in % damage) for categorizing region of interest as damaged or not.

When classifying a patient with ‘phonological impairments’, we are referring to patients who had impaired scores on both the non-word reading and digit span CAT tasks. The severity of phonological impairments was measured by averaging T-scores across the non-word reading and digit span tasks.

### Sample 1

#### Classification accuracy when using regions from previous TMS studies

Overall, the incidence of phonological impairments in patients with above-threshold damage to the SMG, pOp or SMG and pOp regions versus below-threshold damage to both regions was 74% (40/54) and 21% (21/100), respectively. Specifically, we found that the incidence of phonological impairments for damage to (SMG and pOp), (SMG not pOp), and (pOp not SMG) was: 88% (15/17), 75% (12/16) and 62% (13/21), respectively. In those with below-threshold damage to both regions, the incidence of phonological impairments after 20–79% damage versus 0–19% damage to either SMG or pOp was: 41% (11/27) and 14% (10/73), respectively.

#### Classification accuracy when using regions from previous functional MRI studies

Overall, the incidence of phonological impairments was 44/69 (64%) patients with above-threshold damage to the functional MRI regions and 17/85 (20%) in those with below-threshold damage to these regions. As shown in Table 1, the positive predictive value of the TMS regions (74%) was significantly higher than that of the functional MRI regions (64%; *χ*^2 ^= 4.51, *P* = 0.034). This resulted in a lower odds ratio for the functional MRI regions than the TMS regions (7.0 versus 10.7; Table 1), suggesting that the TMS regions provide a better account of the data than the functional MRI regions. Moreover, using both TMS and functional MRI regions did not improve the classification accuracy relative to using only the TMS regions (9.5 versus 10.7; Table 1).

#### Classification accuracy when using the TMS-guided regions

The TMS-guided regions differed from the spherical TMS regions because only the TMS-guided regions extended deep into the underlying white matter, including the anterior, posterior and long segments of the superior longitudinal fasciculus/arcuate fasciculus and the inferior fronto-occipital fasciculus (Fig. 2A and Supplementary Fig. 2). The TMS-guided SMG region additionally included parts of the inferior longitudinal and uncinate fasciculi; while the TMS-guided pOp region additionally included the external and internal capsule.

Overall, the incidence of phonological impairments in patients with above-threshold damage to one or both of the TMS-guided regions versus below-threshold damage to both TMS-guided regions was 85% (46/54) and 15% (15/100), respectively. When we considered the effect of each lesion site separately, we found that the incidence of phonological impairments after damage to (SMG and pOp), (SMG not pOp), and (pOp not SMG) was: 89% (17/19), 90% (9/10) and 80% (20/25), respectively. In those with below-threshold damage to both regions, the incidence of phonological impairments after 20–79% damage versus 0–19% damage to either SMG or pOp was: 21% (13/62) and 5% (2/38), respectively.

**Figure 3:**
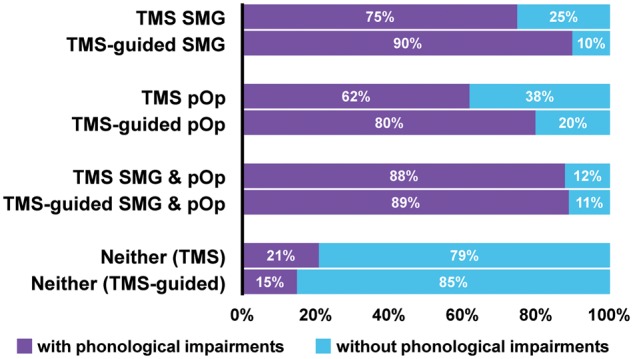
**Classification accuracy for TMS and TMS-guided regions.** Improvements in the classification accuracy can be seen when the lesion categorization changed from original TMS regions to TMS-guided regions. Patients with above-threshold damage to the TMS-guided regions had higher incidence of phonological impairments (i.e. impaired non-word reading and digit span) than patients with above-threshold damage to the original TMS regions.

As expected when data are fitted, the classification accuracy was higher for the TMS-guided regions than the original TMS regions [odds ratio (OR) = 32.6 versus 10.7]. Importantly, however, the fitting of the data was only based on a subset of patients. In patients who were not used to define the TMS-guided regions, 10 had below-threshold damage to both TMS sites but above-threshold damage to the TMS-guided sites (one SMG, eight pOp and one both) (Table 2). The classification accuracy in these 10 patients was higher for the TMS-guided regions (8/10 with damage had phonological impairments) than for the TMS regions (2/10 without damage did not have phonological impairments). Over all 154 patients, the incidence of phonological impairments rose to 85% (46/54) from 74% (40/54) in those with above-threshold damage to the TMS-guided versus TMS regions while falling to 15% (15/100) from 21% (21/100) in those with below-threshold damage to the TMS-guided versus TMS regions (Fig. 3). The classification accuracy of the TMS-guided regions cannot be explained by lesion size because, even when lesion size was matched, the incidence and severity of phonological impairments was significantly worse when either of the TMS-guided regions were damaged compared to spared (Tables 3 and 5).

**Table 2:** Lesion categorization

Damage to:	TMS-guided SMG	TMS-guided pOp	TMS-guided SMG and pOp	Neither
TMS SMG	9 (8)	0	3 (3)	4 (1)
TMS pOp	0	15 (12)	0	6 (1)
TMS SMG and pOp	0	2 (2)	15 (13)	0
Neither	1 (1)	8 (6)	1 (1)	90 (13)

The numbers of patients who moved from one group to another, with number of patients who were impaired on the CAT phonological measure (i.e. combined non-word reading and digit span) shown in parentheses.

**Table 3:** Summary of demographic and clinical data for each TMS-guided group

		**Size matched**			**Excluded from size-matched groups**			
		**SMG**	**pOp**	**Control**	**SMG**	**pOp**	**SMG and pOp**	**Control**
		*n* = 8	*n* = 13	*n* = 30	*n* = 2	*n* = 12	*n* = 19	*n* = 70
Lesion size (cm^3^)	Mean	85.1	90.8	77.6	133.9	176.2	230.2	19.9
	SD	29.2	20.9	19.8	2.5	34.0	83.9	14.8
	Minimum	44.3	57.6	51.5	132.1	132.2	116.5	1.4
	Maximum	117.8	128.7	127.0	135.7	226.0	464.7	50.8
Age (years)	Mean	59.9	50.6	60.3	68.6	56.2	59.7	59.9
	SD	10.2	14.5	12.0	2.8	8.4	12.6	13.4
	Minimum	46.5	30.7	29.4	66.7	40.1	38.9	21.3
	Maximum	78.3	74.1	78.2	70.6	65.1	85.5	90.0
Time post-stroke (months)	Mean	30.3	27.2	32.0	51.3	25.7	42.2	32.1
	SD	15.3	11.3	12.5	6.6	10.5	13.3	14.0
	Minimum	13.5	13.5	12.4	46.6	12.5	14.4	12.0
	Maximum	53.6	45.9	55.6	55.9	50.3	60.0	57.4
Gender	Males	7	9	26	2	10	16	41
	Females	1	4	4	0	2	3	29
Phonological measure	Imp (not)	8 (0)	10 (3)	10 (20)	1 (1)	10 (2)	17 (2)	5 (65)
	Mean	45.1	46.9	53.1	54.0	47.3	42.5	59.4
	SD	4.2	7.0	7.3	4.2	5.8	4.6	6.4
	Minimum	37.5	37.5	37.5	51.0	37.5	37.5	41.5
	Maximum	50.0	60.0	67.0	57.0	58.0	55.5	67.0
% damage to SMG	Mean	93.8	30.8	46.8	100	47.8	93.5	10.4
	SD	6.1	27.3	26.2	0.0	26.8	6.9	16.5
	Minimum	86.0	0.0	0.0	100	3.0	83.0	0.0
	Maximum	100	72.0	79.0	100	78.0	100	76.0
% damage to pOp	Mean	19.8	93.3	33.2	11.0	93.9	95.2	16.3
	SD	23.9	8.2	28.3	15.6	8.0	6.7	22.1
	Minimum	1.0	80.0	0.0	0.0	80.0	80.0	0.0
	Maximum	60.0	100	78.0	22.0	100	100	74.0

All data are from Sample 1 (*n* = 154). The three groups listed on the left-hand side were matched for lesion size [*F*(2,48) = 1.78, *P* = 0.180]. The four groups listed on the right-hand side comprise the patients that were excluded from the size matched groups. Imp = number of patients with impaired phonological scores; not = number of patients who did not meet our criteria for phonological impairments.

#### Classification accuracy when using the functional MRI-guided regions

The functional MRI-guided pOp region was similar, though not identical, to the TMS-guided pOp region (Fig. 2). Consequently, both regions had comparable positive predictive values when considered without SMG (37/44 for TMS-guided pOp and 28/32 for functional MRI-guided pOp). However, more patients were accounted for by the TMS-guided pOp region (37) than the functional MRI-guided pOp region (28). When we also took into account the SMG regions, the explanatory power of the TMS-guided regions increased further (OR = 32.6), but this was not the case for the functional MRI-guided regions (OR = 18.9) (Table 1).

#### Classification accuracy when using the unguided lesion overlap map region

The unguided-LOM region differed from the TMS-guided regions (Fig. 2) because, without using the TMS sites from prior studies to stratify the patients into different groups, the most frequent area of overlap was the white matter between the two TMS regions (see Mah *et al.*, 2014 for illustrations of how and why this happens). This unguided-LOM region included portions of the anterior, posterior and long segments of the superior longitudinal fasciculus/arcuate fasciculus, as well as the periventricular white matter.

Phonological impairments were observed in 33/37 (89%) patients with above-threshold damage to the unguided-LOM region and 28/117 (24%) patients with below-threshold damage to this region. This resulted in a lower odds ratio for the unguided-LOM region (26.2) than the TMS-guided regions (32.6), with a negligible improvement when the analysis used both the unguided-LOM region and the TMS-guided regions (OR = 33.9) (Table 1). The unguided-LOM region did nevertheless have higher classification accuracy than the functional MRI-guided regions (OR = 26.2 versus 18.9) (Table 1).

#### Classification accuracy when using the VLSM region

The VLSM region (where the frequency of lesions is most significantly different in patients with versus without phonological impairments) was identified in the left superior temporal gyrus including portions of the planum temporale and Heschl’s gyrus. This region is completely different from the other regions (Fig. 2), because VLSM takes into consideration the variance in lesion site within and between groups as well as the overlap in lesion site (Rudrauf *et al.*, 2008).

The incidence of phonological impairments was 33/39 (85%) in patients with above-threshold damage to the VLSM region and 28/115 (24%) in patients with below-threshold damage to the VLSM region. The odds ratio for the VLSM region was substantially lower than that of the TMS-guided regions (17.1 versus 32.6) and the unguided-LOM region (17.1 versus 26.2), but only slightly lower than that of the functional MRI-guided regions (17.1 versus 18.9) (Table 1). However, because the VLSM region accounted for seven patients with phonological impairments that did not have above-threshold damage to the TMS-guided regions, and the TMS-guided regions accounted for 20 patients with phonological impairments who did not have above-threshold damage to the VLSM region (Supplementary Table 1), the odds ratio was highest (49.4) when the analysis used both the VLSM region and the TMS-guided regions.

### Sample 2

#### Classification accuracy

In Sample 2, the prevalence of phonological impairments (40/108 = 37%) was approximately the same as in Sample 1 (61/154 = 40%). The key point of interest, however, was the classification accuracy for different regions of interest (Table 1). We found that, even when all the regions were entirely independent of the data, the odds ratio was still substantially higher for the TMS-guided regions (27.6) than the functional MRI-guided regions (5.7), the unguided-LOM region (10.8) or the VLSM region (5.7). Interestingly, the best fit of the data (OR = 39.7) was when the analysis included the unguided-LOM region as well as the TMS-guided regions (Table 1) and this was not improved further by adding the VLSM region (OR = 30.7) or the functional MRI-guided regions (OR = 39.7).

Finally, the independence of Sample 2 from the region selection process allows us to compare the odds ratio for the original TMS regions (spherical volumes in grey matter) with that of the TMS-guided regions (that extended deep into the white matter). This confirmed that the classification accuracy was substantially better when using the TMS-guided regions than the original TMS regions (OR = 27.6 versus 8.2).

### The effect of damage to the TMS-guided regions on writing heard words

As expected, damage to either of the TMS-guided regions did not differentially affect the incidence of phonological impairments (i.e. impaired non-word reading and digit span) and the incidence of impaired performance on the writing heard words task that involved covert phonological processing without speech production (Tables 4 and 5). However, test scores were, overall, significantly lower on the combined non-word reading and digit span measure than the writing heard words task (Tables 4 and 5); plausibly because accessing the semantic representations of the familiar words lessen the demands on phonological processing.

**Table 4:** Functional impairments after damage to the TMS-guided SMG or pOp regions

**Measure**		**SMG**	**pOp**	**Control**
		*n* = 10	*n* = 25	*n* = 100
Phonological (non-word reading and digit span)	Number of patients imp (not)	9 (1)	20 (5)	15 (85)
	Mean score across group	46.9	47.1	57.5
	SD	5.5	6.3	7.3
Writing heard words (Writ-H_W_)	Number of patients imp (not)	7 (3)	20 (5)	34 (65)^a^
	Mean score across group	52.8	52.1	60.1
	SD	9.3	8.2	7.2
Visual word-to-picture matching (V_W_-P)	Number of patients imp (not)	5 (5)	17 (8)	28 (72)
	Mean score across group	54.9	52.4	58.1
	SD	7.0	6.4	6.7
Auditory word-to-picture matching (A_W_-P)	Number of patients imp (not)	5 (5)	10 (15)	12 (88)
	Mean score across group	53.6	53.4	58.3
	SD	8.1	6.4	5.8
Semantic associations (Sem-A)	Number of patients imp (not)	1 (7)^a^	2 (22)^a^	7 (90)^a^
	Mean score across group	56.4	56.3	57.0
	SD	7.3	5.9	5.5

All data are from Sample 1. The table shows the numbers of patients who had impairments (or did not meet the criteria for impairments) on five different measures that differentially require phonological and semantic processing. imp = patients with impaired phonological scores; not = patients who did not meet our criteria for phonological impairments. ^a^Missing scores: one control for Writ-H_W_, and two SMG, one pOp and three controls for the Sem-A task.

**Table 5:** Statistical analyses

		Incidence (chi-square test)				Severity (*t*-test)			
	*n*	*χ*^2^	*df*	*OR*	*P*	*t*	*df*	*d*	*P*
**Comparison of the incidence and severity of phonological impairments in patients with damage to one of the TMS-guided regions relative to size-matched controls (see also **Table 3**)**									
TMS-guided SMG	8 versus 30	^a^	^a^	^a^	**0.001**	2.91	36	1.16	**0.006**
TMS-guided pOp	13 versus 30	6.93	1	6.67	**0.008**	2.56	41	0.85	**0.014**
**Comparison of the incidence and severity of impairments on the phonological measure relative to that on each of the control tasks in patients with damage to one of the TMS-guided regions (see also **Table 4**)**									
TMS-guided SMG:									
Phon versus Writ-H_W_	10	2.00^b^	1	–	0.157	2.45	9	0.77	**0.037**
Phon versus V_W_-P	10	4.00^b^	1	–	**0.046**	3.69	9	1.17	**0.005**
Phon versus A_W_-P	10	4.00^b^	1	–	**0.046**	2.25	9	0.71	0.051
Phon versus Sem-A	8	6.00^b^	1	–	**0.014**	3.19	7	1.13	**0.015**
TMS-guided pOp:									
Phon versus Writ-H_W_	25	0.00^b^	1	–	1.000	4.73	24	0.95	**<0.001**
Phon versus V_W_-P	25	0.82^b^	1	–	0.366	2.95	24	0.59	**0.007**
Phon versus A_W_-P	25	8.33^b^	1	–	**0.004**	4.10	24	0.82	**<0.001**
Phon versus Sem-A	24	15.21^b^	1	–	**<0.001**	5.32	23	1.09	**<0.001**
**Comparison of the within-subject difference between phonological and semantic scores in patients with damage to one of the TMS-guided regions relative to controls (see also **Table 4**)**									
TMS-guided SMG:									
Phon − V_W_-P	10 versus 100	–	–	–	–	2.82	108	0.93	**0.006**
Phon − A_W_-P	10 versus 100	–	–	–	–	2.18	108	0.72	**0.031**
Phon − Sem-A	8 versus 97	–	–	–	–	3.29	103	1.21	**0.001**
TMS-guided pOp:									
Phon − V_W_-P	25 versus 100	–	–	–	–	2.58	123	0.58	**0.011**
Phon − A_W_-P	25 versus 100	–	–	–	–	3.12	123	0.70	**0.002**
Phon − Sem-A	24 versus 97	–	–	–	–	5.72	119	1.30	**<0.001**

Phon = combined non-word reading and digit span; Writ-H_W_ = writing heard words; V_W_-P = visual word-to-picture matching; A_W_-P = auditory word-to-picture matching; Sem-A = semantic associations; *d* = Cohen’s *d*.

^a^Fisher’s exact test. ^b^McNemar’s chi-square test.

### The effect of damage to the TMS-guided regions on semantically demanding tasks

Crucially, patients with damage to either of the TMS-guided regions performed worse on phonologically demanding tasks than semantically demanding tasks (Tables 4 and 5); and these differences in task performance were significantly larger than those observed in patients with other lesions (i.e. below-threshold damage to both regions) (Table 5). These lesion-specific task effects suggest that the relative sparing of semantic compared to phonological processing abilities in patients with damage to the TMS-guided SMG or pOp regions was not merely driven by differences in task difficulty.

### Differences in the effect of damage to supramarginal gyrus or pars opercularis

Performance on all 27 subtests from the CAT was compared in patients with lesions to the TMS-guided SMG region (*n* = 8) versus TMS-guided pOp region (*n* = 13) after matching for lesion size (Table 3). There were no statistically significant differences in the incidence or severity of impairments after Bonferroni correction for multiple comparisons (Supplementary Fig. 3). Without Bonferroni correction, non-word repetition scores were lower after damage to SMG (mean = 43.8, SD = 7.2) than pOp [mean = 54.5, SD = 8.2; *t*(19) = 3.039, *P* = 0.007], but there were no other statistically significant group differences for any of the tasks.

## Discussion

In this study we have shown how the effect of functionally localized TMS-induced transient lesions in healthy participants guided the identification of lesion sites that caused persistent phonological impairments after stroke. Below, we discuss: how damage to regions from prior TMS and functional MRI studies affects phonological processing; the lesion sites that best explain the incidence of phonological impairments after stroke; and the functional impairments caused by SMG or pOp damage.

### How damage to regions from prior TMS and functional MRI studies affects phonological processing

Our regions of interest were 0.5 cm^3^ spheres centred on MNI coordinates from previous TMS and functional MRI studies of phonological processing. The TMS sites were not the same as the functional MRI sites (Fig. 2) because they had been functionally localized to the position where TMS impaired phonological decisions (Gough *et al.*, 2005; Sliwinska *et al.*, 2015). We found that lesions to the TMS regions accounted for the incidence of persistent phonological impairments better than lesions to the functional MRI regions and that the explanatory power of the TMS sites was not improved by adding the functional MRI sites (Table 1). This suggests that TMS-based functional localization in healthy participants helped to identify which parts of pOp and SMG were necessary for phonological processing.

Although the TMS sites were more predictive than the functional MRI sites, the incidence of phonological impairments after damage to the TMS regions (i.e. the positive predictive value) was still below 80%. This between-subject inconsistency in the mapping between lesion and impairment might reflect (i) intersubject variability in the ability to recover phonological processing skills after damage to the TMS regions; (ii) intersubject variability in the brain regions that support phonological processing; (iii) the sensitivity of our phonological measure (based on the accuracy of non-word reading and digit span) being lower than that used in the TMS studies (i.e. response times to phonological decisions tasks); or (iv) the use of suboptimal regions that do not include the full extent of grey and white matter that is necessary for phonological processing. We pursue the selection of the most optimum regions below, while noting that intersubject variability is also likely to play an important role, as shown in prior TMS (Sliwinska *et al.*, 2015), functional MRI (Seghier and Price, 2016) and continuous theta burst stimulation (Hartwigsen *et al.*, 2013) studies of healthy participants, in addition to studies of recovery after stroke (Forkel *et al.*, 2014).

### The lesion sites that best explain the incidence of phonological impairments

Having tested how phonological processing abilities are affected by damage to regions identified in previous studies, we sought to adapt the regions until they provided a better explanation of which patients did and did not have phonological impairments. This involved tailoring the regions of interest to areas that were most consistently damaged in patients with phonological impairments. We generated four different types of data-driven regions using (i) two TMS-guided LOMs; (ii) a functional MRI-guided LOM; (iii) an unguided LOM; and (iv) VLSM, which identified an area in the left superior temporal cortex that was not part of the TMS-guided or unguided LOMs.

We found that the presence or absence of phonological impairments was explained by the TMS-guided regions better than any of the other sets of regions (functional MRI-guided regions, unguided-LOM region or VLSM region) (Table 1). However, the best fit of the data was when the analysis took into account the VLSM region (in superior temporal cortex) as well as the TMS-guided regions (pOp and SMG). This is because damage to the TMS-guided regions explained phonological impairments in 20 patients who did not have damage to the VLSM regions; while, conversely, damage to the VLSM region explained phonological impairments in seven patients who did not have damage to the TMS-guided regions (see Supplementary material for full breakdown). Together the TMS-guided and VLSM regions were able to account for the incidence of phonological impairments after stroke with nearly 90% sensitivity and specificity (Table 1). This is quite remarkable given that the phonological impairments were defined using different tasks and measures (accuracy versus response times) than those used in the previous TMS studies. Moreover, our lesion-deficit associations provide a much more consistent explanation of the incidence of aphasic symptomatology than that expected on the basis of recent reviews (Watila and Balarabe, 2015) and may, in future, help to improve our ability to predict outcome after stroke.

By additionally including a second sample of patients (those who were >5 years post-stroke) we were also able to show that lesions to the TMS-guided regions explained phonological impairments (in a new sample) better than lesions to the original TMS regions. This indicates that phonological impairments were, in part, caused by damage to the white matter underlying the original TMS regions, and it raises the possibility that the effect of TMS in healthy participants might also be emerging from disruption to much more extensive areas than the recorded site of stimulation. Indeed, prior studies have already claimed that the effect of TMS is not limited to the cortical mantle but spreads through the network (Siebner *et al.*, 2009; Wagner *et al.*, 2009; Nummenmaa *et al.*, 2014). Further analyses of lesion sites are needed to determine whether phonological impairments result from damage to the cortex only, underlying white matter only or both. The results might also offer new insights into how TMS affects phonological processing in healthy participants.

In summary, prior TMS studies have shown that pOp and SMG contribute to the speed of phonological processing but were not able to test whether these regions are essential for generating accurate responses. The novel contribution of the current study is to identify the extent of pOp and SMG damage that impairs accurate phonological processing, even years after stroke. Our findings demonstrate that the pOp and SMG regions we have identified play a unique role in phonological processing that cannot typically be subsumed by other brain areas.

### The functional impairment caused by supramarginal gyrus and pars opercularis damage

The observation that damage to our TMS-guided SMG and pOp regions impaired phonological more than semantic tasks mirrors prior findings from TMS (Gough *et al.*, 2005; Sliwinska *et al.*, 2015) and functional MRI (Devlin *et al.*, 2003; McDermott *et al.*, 2003) studies of healthy participants. Likewise, the fact that damage to our TMS-guided SMG and pOp regions impaired the ability to write words to dictation is consistent with previous studies associating SMG and pOp with covert rather than overt phonological processing (Crottaz-Herbette *et al.*, 2004; Gough *et al.*, 2005; Wimmer *et al.*, 2010; Geva *et al.*, 2011; Sliwinska *et al.*, 2015).

We did not find any evidence that lesions to the SMG or pOp regions had differential effects on behaviour, despite comparing patient performance on a set of tasks that systematically varied the demands on a wide range of sensory, motor and cognitive functions. Nor did we find any indication that concurrent damage to our SMG and pOp regions impaired phonological processing more than damage to each region alone. This finding mirrors that observed in TMS studies of healthy participants (Hartwigsen *et al.*, 2016), suggesting that SMG and pOp form part of the same phonological processing system, which breaks down following damage to any critical part.

## Conclusion

In conclusion, we have shown that: (i) findings from TMS studies of healthy participants helped to identify lesion sites that explained phonological processing abilities after stroke; (ii) damage to the TMS-guided SMG and pOp regions affected performance on phonological more than semantic tasks; (iii) the stroke lesions impacting upon phonological abilities extended, in the majority of cases, deep into the white matter; and finally, (iv) most patients with lesions to these extended SMG and pOp regions incorporating both grey and white matter were not able to recover their phonological processing abilities even years after stroke. The TMS-guided regions we have identified therefore appear to be critical for normal phonological processing.

## Supplementary Material

Supplementary Data

## Abbreviations

CAT: Comprehensive Aphasia Test
LOM: lesion overlap map
pOp: pars opercularis of the left inferior frontal gyrus
SMG: left supramarginal gyrus
TMS: transcranial magnetic stimulation
VLSM: voxel-based lesion-symptom mapping
