# Supplementary material for: Using transcranial magnetic stimulation of the undamaged brain to identify lesion sites that predict language outcome after stroke
Source: Brain. 2017 Apr 18;140(6):1729–42. doi: 10.1093/brain/awx087 (PMC5445259; doi:10.1093/brain/awx087)
Supplement: Supplementary Data [file awx087_Supp.pdf]

Supplementary Table 1: Incidence of phonological impairments according to lesion site

**Above-threshold damage to:**	**Phonological impairments?**	
	Yes	No
(a) Sample 1 (1-5 years post-stroke), ROIs from prior studies (independent of data)		
TMS & fMRI	35	11
TMS only	5	3
fMRI only	9	14
Other lesion sites	12	65
Error	**20%**	**30%**
(b) Sample 1 (1-5 years post-stroke), data-driven ROIs		
TMS-guided, fMRI-guided, LOM & VLSM	18	2
TMS-guided, fMRI-guided & LOM not VLSM	4	0
TMS-guided, fMRI-guided & VLSM not LOM	1	0
TMS-guided, LOM & VLSM not fMRI-guided	5	0
TMS-guided & fMRI-guided not LOM & VLSM	4	2
TMS-guided & LOM not fMRI-guided & VLSM	3	0
TMS-guided & VLSM not fMRI-guided & LOM	2	1
LOM & VLSM not TMS-guided & fMRI-guided	3	1
TMS-guided only	9	3
fMRI-guided only	1	0
LOM only	0	1
VLSM only	4	2
Other lesion sites	7	81
Error	**11%**	**13%**
(c) Sample 2 (> 5 years post-stroke), ROIs from Sample 1 (independent of data)		
TMS-guided, fMRI-guided, LOM & VLSM	11	2
TMS-guided, fMRI-guided & LOM not VLSM	7	5
TMS-guided, fMRI-guided & VLSM not LOM	1	1
TMS-guided, LOM & VLSM not fMRI-guided	5	1
TMS-guided & fMRI-guided not LOM & VLSM	3	4
TMS-guided & LOM not fMRI-guided & VLSM	2	1
TMS-guided & VLSM not fMRI-guided & LOM	3	2
LOM & VLSM not TMS-guided & fMRI-guided	1	1
TMS-guided only	5	5
fMRI-guided only	0	0
LOM only	0	0
VLSM only	0	4
Other lesion sites	2	42
Error	**5%**	**38%**

The error term represents the percentage of patients with phonological impairments in the absence of ROI damage (i.e. false negative rate; left-hand side column) as well as the percentage of patients who did not meet the criteria for phonological impairments in the presence of ROI damage (i.e. false positive rate; right-hand side column). Combinations of lesion sites that did not exist in the data are not included in the table (e.g., fMRI-guided, LOM & VLSM not TMS-guided).

Supplementary Figure 1: Task analysis

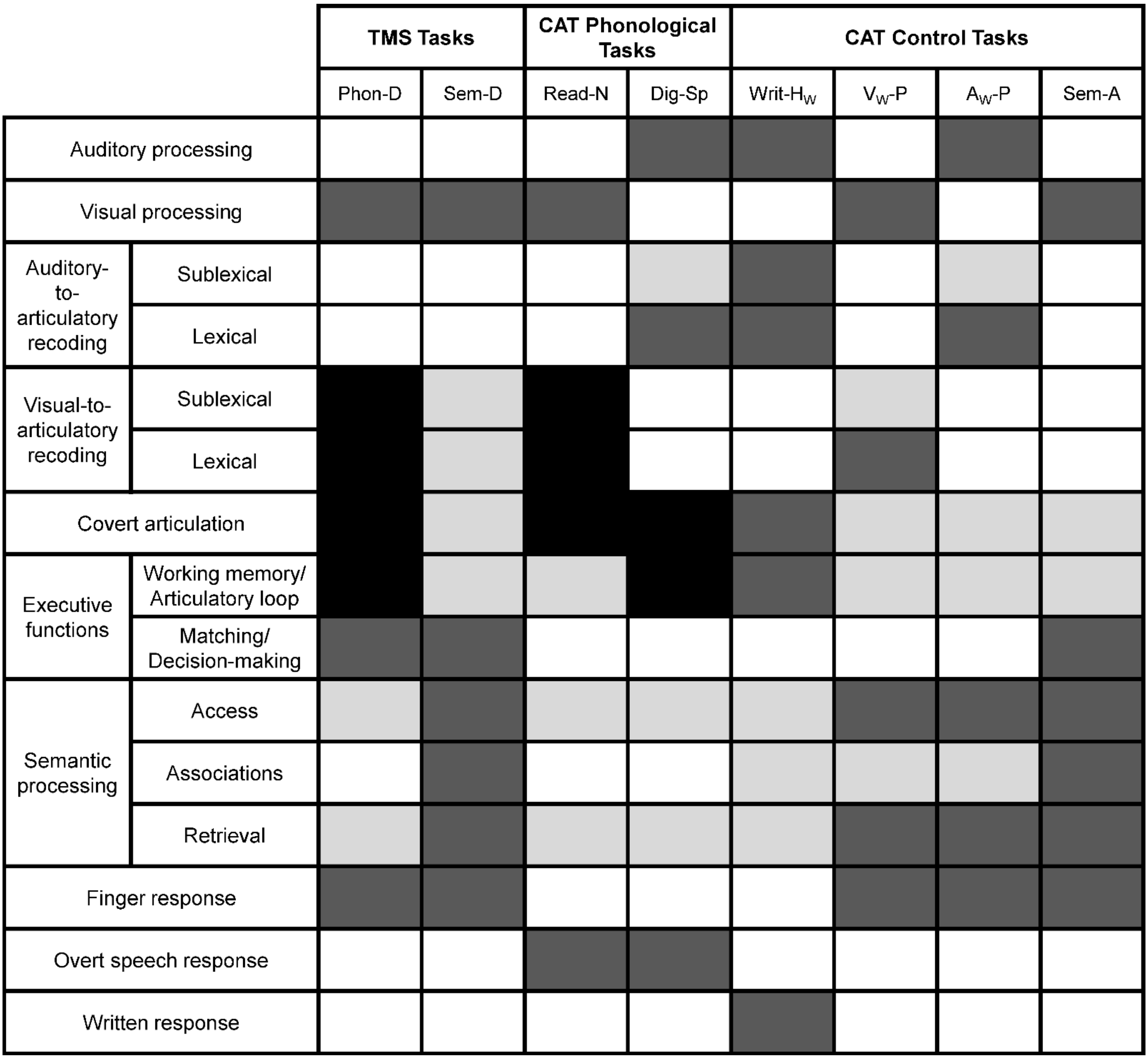

The levels of processing hypothesized to be required for completing the TMS phonological (Phon-D) and semantic (Sem-D) decision tasks, and the following tasks from the CAT: nonword reading (Read-N), digit span (Dig-Sp), writing heard words (Writ-H_W), visual word-to-picture matching (V_W-P), auditory word-to-picture matching (A_W-P), and semantic associations (Sem-A). Black is used to highlight the phonological processes of interest that are shared by the TMS phonological task and at least one of the CAT phonological tasks. Dark grey indicates necessary/explicit processes. Light grey signifies supporting/implicit processes.

Supplementary Figure 2: Lesion overlap maps

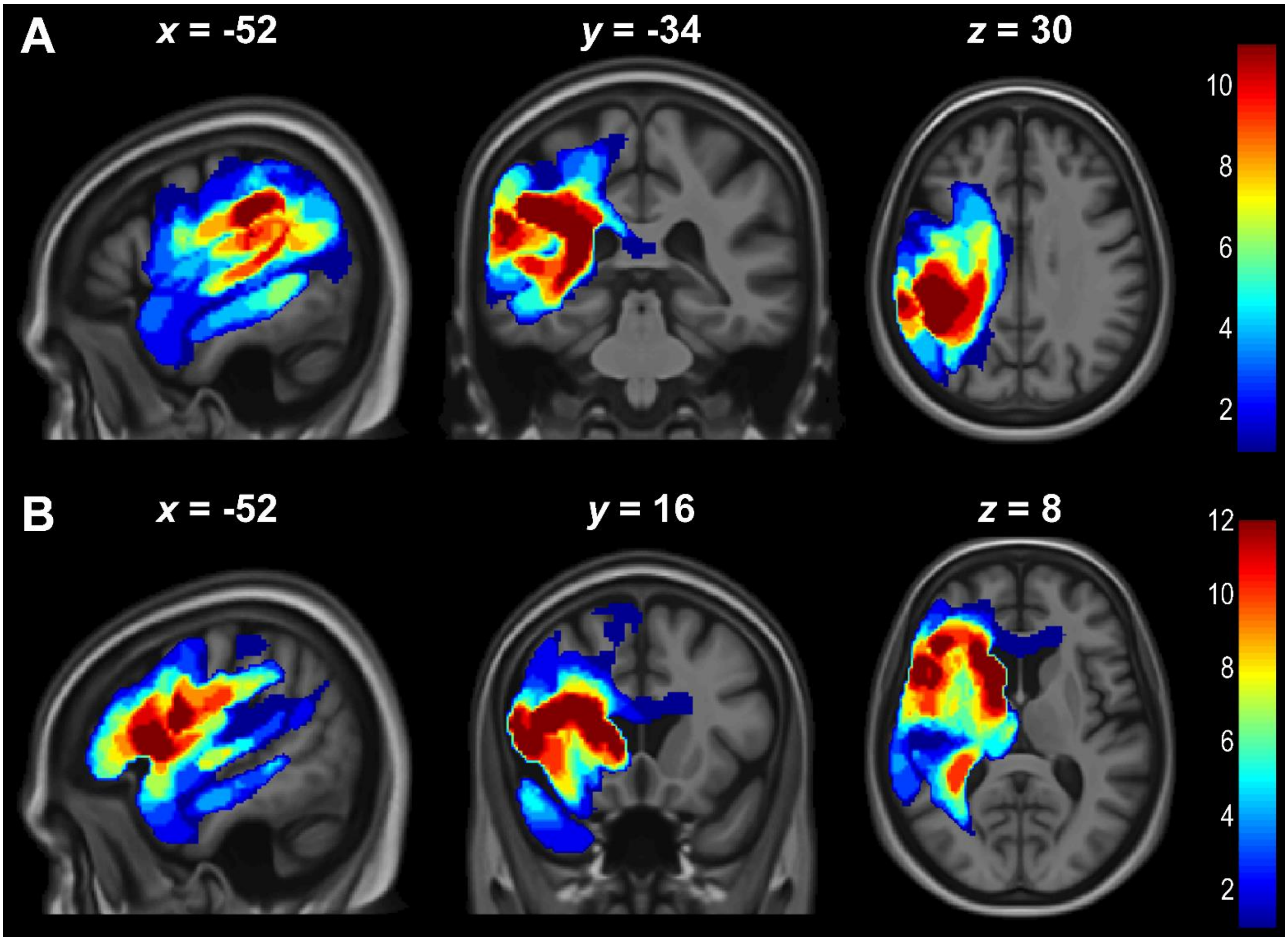

(A) Lesion overlap map of patients with phonological impairments and above-threshold damage to the TMS SMG region (n = 11). The TMS-guided SMG region comprised voxels that were damaged in at least 10 out of 11 patients from the lesion overlap map. **(B)** Lesion overlap map of patients with phonological impairments and above-threshold damage to the TMS pOp region (n = 12). The TMS-guided pOp region comprised voxels that were damaged in 12 out of 12 patients from the lesion overlap map.

Supplementary Figure 3: The effect of lesion site on performance of 27 subtests from the CAT

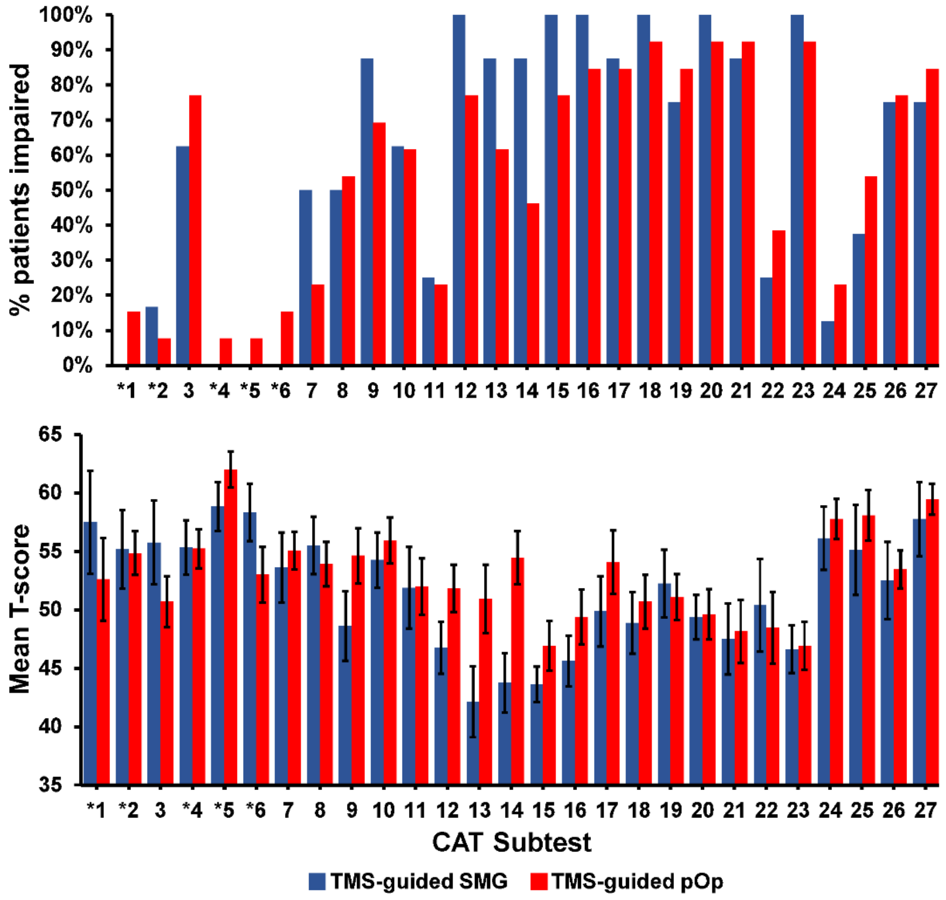

A key to the abbreviations:

1 = Line bisection
2 = Semantic associations
3 = Word fluency
4 = Recognition memory
5 = Gesture object use
6 = Arithmetic
7 = Auditory word-to-picture matching
8 = Visual word-to-picture matching
9 = Auditory sentence-to-picture matching
10 = Visual sentence-to-picture matching
11 = Spoken paragraph comprehension
12 = Word repetition
13 = Complex word repetition
14 = Nonword repetition
15 = Digit span
16 = Sentence repetition
17 = Object naming
18 = Action naming
19 = Spoken picture description
20 = Word reading
21 = Complex word reading
22 = Function word reading
23 = Nonword reading
24 = Writing: Copying
25 = Writing picture names
26 = Writing heard words
27 = Written picture description

Top row: plot of the incidence of impairments. Bottom row: plot of the severity of impairments. On the right-hand side, a key to the abbreviations used in the figure is provided. * = two patients with above-threshold damage to the TMS-guided SMG region did not complete these tasks from the CAT.
